# 614. Experience with Using MALDI-TOF MS to Diagnose Clinically Significant Filamentous Fungal Isolates

**DOI:** 10.1093/ofid/ofad500.680

**Published:** 2023-11-27

**Authors:** Hugh Murray, Amy Crowe

**Affiliations:** St Vincent's Hospital Melbourne, Melbourne, Victoria, Australia; St Vincent's Hospital Melbourne, Melbourne, Victoria, Australia

## Abstract

**Background:**

Matrix assisted laser desorption/ionisation time of flight mass spectrometry (MALDI-TOF MS) technology has revolutionised bacterial diagnostics. Filamentous fungi are a varied and important group of pathogens that cause a wide spectrum of clinical disease. Identification of these traditionally relies upon highly experienced practitioners identifying unique fungal morphological characteristics, along with specific molecular techniques, but MALDI-TOF MS may be a useful alternative. The aim of this retrospective review was to determine how useful MALDI-TOF MS was as an adjunct to filamentous fungi identification at our laboratory service.

**Methods:**

All fungal diagnoses made at St Vincent’s Pathology Melbourne, a large metropolitan laboratory servicing multiple tertiary hospitals and community pathology centres, from 2018 to 2022 were extracted. For clinically significant filamentous fungal isolates, the method of diagnosis was reviewed and categorised into; morphological diagnosis, Bruker MALDI Biotyper® diagnosis, and national reference laboratory diagnosis. All clinically significant isolates where MALDI-TOF MS was attempted were also reviewed.

**Results:**

A total of 256 clinically significant isolates were extracted and reviewed. MALDI-TOF MS was attempted in 59 of these, with success in 39 (66.1%). Of the 20 cases where MALDI-TOF MS was attempted but did not identify the isolate, 16 were diagnosed morphologically and four required send out to the national reference laboratory. 71.2% of MALDI-TOF MS attempts were in the 2021-22 period when successful identification occurred 80.9% of the time. No species/genus appeared more or less readily identifiable. Of the remaining cases, 208 were identified morphologically and nine using the national reference laboratory.

**Conclusion:**

MALDI-TOF MS performed modestly well at identifying filamentous fungi during this study period. Increased use and successful isolate identification in the 2021-22 period likely reflects improvement in the Bruker MALDI-TOF MS database for filamentous fungi. The MALDI-TOF MS system is a useful option in the identification of clinically significant filamentous fungi however morphological and molecular techniques remain important.

**Disclosures:**

**All Authors**: No reported disclosures

